# Colorectal cancer at the anastomotic site following childhood surgery for hirschsprung disease: a rare case report

**DOI:** 10.1186/s12957-025-03754-w

**Published:** 2025-03-28

**Authors:** Matthias Mehdorn, Philipp Rhode, Jan-Hendrik Gosemann, Katja Grunwald, Hans-Jonas Meyer, Martin Lacher, Sigmar Stelzner

**Affiliations:** 1 Department of Visceral, Transplant, Thoracic and Vascular Surgery, University Hospital of Leipzig, Liebigstraße 20, 04103 Leipzig, Germany; 2 Department of Pediatric Surgery, University Hospital Leipzig, Leipzig, Germany; 3 Gastroenterologische Praxis am Johannisplatz, Leipzig, Germany; 4 Department of Diagnostic and Interventional Radiology, University Hospital Leipzig, Leipzig, Germany

**Keywords:** Hirschsprung disease, Rectal cancer, Duhamel procedure, Intersphincteric resection, Case report

## Abstract

**Background:**

The present case of a colorectal adenocarcinoma at the anastomotic site of a colorectal anastomosis after childhood surgery for Hirschsprung disease is a rare report of such pathology. Possibly, the altered anatomy after Duhamel procedure may pose a risk for carcinogenesis in those patients. The previously surgically opened plane of the mesorectal fascia, which is usually dissected during total mesorectal excision in rectal cancer, and the impossibility to differentiate from colon or rectal cancer in this case influence oncologic treatment strategies. Furthermore, the case highlights functional aspects of lower anterior resection syndrome before and after rectal cancer surgery with a coloanal anastomosis and how this influences quality of life.

**Case presentation:**

We report a rare case of a 54-year-old male with a history of Hirschsprung disease, treated with a Duhamel procedure during childhood, who developed colorectal cancer at the site of the colorectal anastomosis. The Duhamel procedure, a common surgical technique in Hirschsprung disease, involves creating a deep colorectal anastomosis with a retained rectal stump. The tumor, a moderately differentiated adenocarcinoma, was treated with total mesorectal excision and intersphincteric resection with a hand-sewn coloanal anastomosis and a loop ileostomy. Postoperative complications included paralytic ileus and urinary retention, but the ileostomy was successfully reversed. Despite increased LARS (lower anterior resection syndrome) scores postoperatively, the patient adapted well, reporting minimal impact on quality of life. In the short term of 1.5 years post-surgery, the patient is disease-free.

**Conclusion:**

This case highlights the need for awareness of potential colorectal cancer development in patients with a history of Hirschsprung disease and emphasizes the importance of individualized management and close surveillance strategies.

## Background

Colorectal carcinoma is a top three cancer in western countries in both women and men. Routine diagnostics include colonoscopy with biopsies. While colon cancer without distant metastasis may be treated with primary surgery, rectal carcinoma of the middle and lower third mostly are subject to multimodal treatment [1]. Oncologic colorectal surgery adheres to strict principles, using embryologic planes for mobilization of the visceral package, i.e. the total mesorectal excision as described by Heald. These techniques have led to improved oncologic outcomes for colon as well as rectal cancers [2, 3].

Hirschsprung disease is a congenital condition affecting the colon and rectum, characterized by the absence of enteric nerve cells (aganglionosis) and presenting with bowel obstruction, primarily in neonates. Initial management often includes creating a diverting ostomy to relieve bowel obstruction until a diagnosis is confirmed. Definitive treatment involves surgical resection of the aganglionic segment of the colon.

Several different techniques have been suggested over time. In the 1950s, Bernhard Duhamel introduced a procedure for Hirschsprung disease that involved resecting the aganglionic colon segment, transecting the rectal stump, and performing a blunt retrorectal dissection to enable a retrorectal pull-through of the colon. The dorsal anorectal junction was divided, and the retrorectal plane was accessed through the external sphincter fibers. An initial end-to-side colorectostomy was created by suturing and clamping the rectal and colon walls, followed by the final anastomosis a week later [4]. Alternatively, the Soave and Swenson technique involve an anal pull-through of the colon with a transanally sutured anastomosis. The Soave technique preserves a muscular cuff, while the Swenson technique includes a full-thickness resection down to the sphincter muscle. Studies have explored the advantages and drawbacks of these methods, with both carrying a risk of incontinence, soiling, or constipation [5, 6]. In the past, Duhamel’s procedure was linked to better quality of life [7], especially if a short stump and a deep anastomosis are considered [8]. Although Hirschsprung disease may be associated with RET- oncogene mutations leading to endocrine neoplasms [9], a recent study found no evidence of an increased overall cancer risk, particularly colorectal cancer [10]. However, as the mean age at follow-up in that study was 19 years, this may be too young to detect potential colorectal cancer development. To date, little is known on the long-term oncologic outcomes of the procedures, as most studies have focused on functional outcomes. Only few cases of colorectal cancer following surgery for Hirschsprung disease have been reported. This raises the question of whether the surgical procedure itself may constitute a risk factor for colorectal cancer at the anastomotic site. Furthermore, it remains to be determined whether subsequent treatment strategies should be adapted to align with current oncologic guidelines.

The case report highlights the challenges and key considerations in managing a patient that developed a colorectal cancer at the anastomotic site after childhood surgery for Hirschsprung disease.

## Case presentation

We report the case of a 54-year-old male who underwent diverting ostomy at the age of four years, followed by a subsequent bowel resection a few months later. The exact medical history was unclear, but he reported having been diagnosed with Hirschsprung disease. Five years prior to the presentation in our department, he suffered from small bowel obstruction, which was managed with an explorative laparotomy. During the procedure, multiple interenteric adhesions were successfully separated, restoring intestinal passage. He fully recovered from that procedure without any further symptoms.

**Fig. 1:**
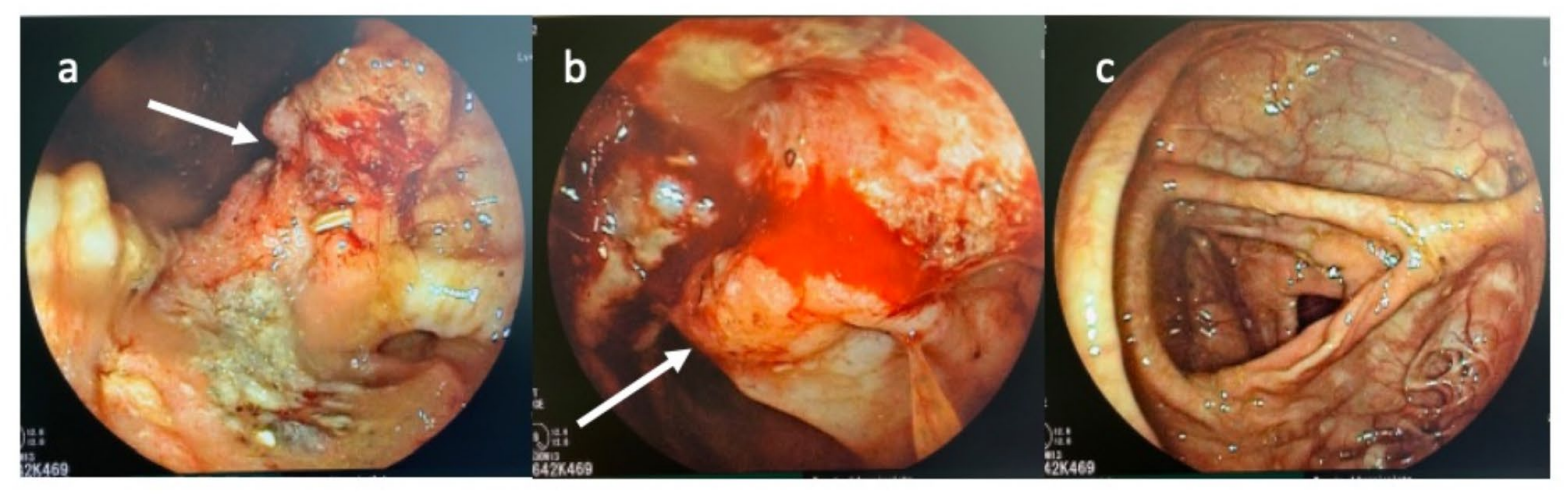
Endoscopic findings during colonoscopy. Images **a** and **b** show the tumorous lesion from different angles (white arrow). The white arrow covers the rectal opening. Image **c** shows the colon limb with scar tissue at the right border

He usually would see his gastroenterologist for routine follow-ups due to the bowel resection in the childhood with the last colonoscopy three years prior yielding no pathology. Now he was discovered with positive occult blood stool test and consequently received a full colonoscopy which revealed neoplastic tissues supraanally at the dorsal aspect of the colorectal anastomosis (Fig. 1a-c). Biopsies showed moderately differentiated adenocarcinoma with no microsatellite instability, other routine parameters such as BRAF and KRAS/NRAS were wildtype. He had no known family history for malignant diseases.

**Fig. 2:**
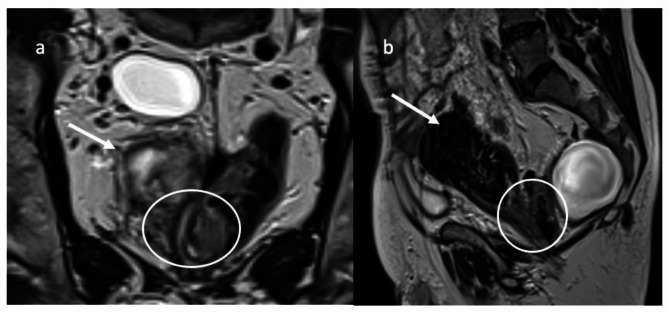
MRI images from the staging. (**a**) coronal T2, (**b**) sagittal T2 imaging. The white arrow points at the massively dilated rectal stump with fecal remnants. The white circles point at the anastomotic region

**Fig. 3:**
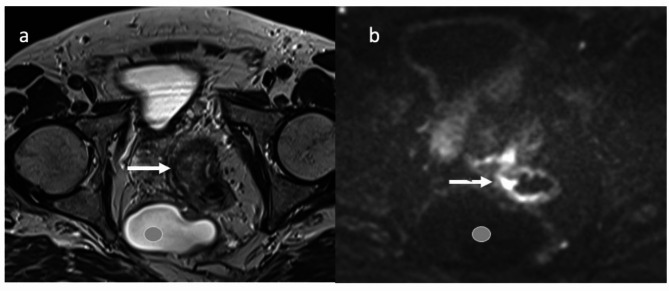
**a** Axial T2 images of the presacral cyst (grey circle) and the tumor at the dorsal circumference of the anastomosis. **b** Diffusion weighted MRI images at the same height of a with intense signal in the tumor at the anastomotic region

Subsequently, he presented in our specialized colorectal cancer unit where rectoscopy revealed a rectal stump measuring nearly ten centimeters. The retrorectal colon anastomosis was difficult to intubate due to the anguliation. Stool remnants were trapped in the rectal stump (Fig. 2). The tumor was located three centimeters from the anal verge. CT imaging excluded distant metastases. MRI imaging showed a cT2N0 tumor, with a diameter of 3.2 cm, no suspicious lymph nodes, negative circumferential resection margin and no extramural vascular invasion. Additionally, a presacral cyst was present that showed no signs of malignancy (bright T2 hyperintense signal without nodular changes or diffusion restriction) (Fig. 3). The interdisciplinary tumor board recommended primary resection in accordance with German guidelines for colorectal cancer [1]. Due to the previous surgeries, the procedure was undertaken as laparotomy, beginning with adhesiolysis, which required one hour to complete. The total mesorectal excision (TME) was initiated by ligating the inferior mesenteric artery (low tie) and vein. Hereafter, the descending colon was mobilized and the mesorectal plane could be reached although its identification was impeded by the previously performed procedure in this plane and the rectal stump was identified at the pelvic inlet. The autonomous nerves were succesfully visualized and preserved. The presacral cyst was dissected en bloc with the specimen, as it exhibited no macroscopic connections to surrounding tissues. Following transection of the colon, a transanal intersphincteric resection was performed and a side-to-end coloanal anastomosis was sewn. Finally, a diverting ileostomy was placed. The postoperative course was delayed by a paralytic ileus as well as urinary retention, necessitating transurethral catheterization. The histopathologic specimen revealed a pT2N0 tumor, leading to structured follow-ups. The ileostomy could be reversed after three months. The urinary retention remained unchanged despite sacral nerve stimulator testing. Therefore, the urinary retention was most likely caused by a neurogenic acontractile detrusor subsequent to intraoperative autonomous nerve lesion. The patient since performs intermittent self-catheterization. In a follow-up visit 18 months after surgery, the patient reported a LARS Score of 41 (major LARS) with urgent defecations and high defecation frequencies but with no soiling. Although he had denied major continence issues prior to the treatment, he later admitted having had defecation issues corresponding to a LARS Score of 27 (minor LARS).

## Discussion

We present a rare case of anastomotic carcinoma of the rectum in a patient that had been operated for Hirschsprung disease during childhood. The case poses several challenges, which we will outline in the following discussion.

Hirschsprung disease is a very rare condition with an incidence of 1 in 5000 live births and only some hundred procedures are performed in Germany annually [11]. The procedure is typically performed in specialized pediatric colorectal surgical centers, as transanal anastomosis in neonates requires significant expertise and advanced technical skills. For the adult gastroenterologist and colorectal surgeon those patients are rarely encountered. In particular, the anatomy after Duhamel’s procedure with the coexistence of a rectal stump and an ultra-deep colorectal anastomosis are uncommon. In contrast, the anatomy after Soave and Swenson procedures resembles the regular anatomy after a colorectal anastomosis. Thus, the first major challenge lies in understanding the anatomy and interpreting colonoscopy and MRI images in these patients.

The location of the tumor and, consequently, its origin whether from the rectum or the colon, may raise controversy as treatment strategies may differ: Locally limited colon cancer is typically treated with primary resection, whereas rectal cancer in the lower third may often be treated with neoadjuvant radio or radio-chemo therapy to improve local recurrence rates [1]. From the MRI imaging and the endoscopy, the tumor in our patient did not clearly originate from the rectal nor the colon limb but in the end, it was treated and classified as a rectal cancer. No neoadjuvant treatment was performed, as it was a localized tumor with no invasion of the mesorectum (cT2) and no other risk factors for metastatic spread or local recurrence. From an oncologic point of view, managing such a tumor in accordance with rectal cancer treatment guidelines appears to be a reasonable approach.

The mesorectal plane may be compromised after previous surgeries to the rectum. Yielding a correct oncologic specimen during total mesorectal excision (TME) is crucial for improveing oncologic outcomes with low local recurrence rates [3]. During Duhamel’s procedure the rectum is mobilized dorsally within mesorectal plane, potentially altering its lymphatic drainage with an increased risk for local recurrence in lateral lymph nodes or a locally unusual spreading pattern. No intact circumferential resection margin exists because of the interrupted mesorectal plane by the afferent colon limb. Nonetheless, in the present case, the mesorectal plane could be used to ensure a TME, despite the challenges posed by the prior surgery. However, the dissection was challenging and supposedly led to autonomous nerve damage with subsequent bladder dysfunction.

Postoperative continence needs to be discussed in a case with several rectal surgeries that may lead to diminished defecation function due to the lacking continence organ, i.e. lower anterior resection syndrome (LARS). Besides deep rectal anastomosis reoperation is also a factor that might promote LARS [12]. Although the LARS score provides a tool to estimate the continence function after rectal surgery, its values do not necessarily correlate with quality of life [13]. Apparently, our patient had LARS prior to the cancer surgery, but that did not limit his overall well-being. After surgery, he reported higher LARS values. As he was accustomed to defecation problems from his childhood days, he still did not consider major LARS to be of concern and he developed strategies to cope with it early after surgery.

The location of the tumor in this case suggests a potential correlation between the Duhamel procedure and colorectal cancer. Literature research via pubmed, medline and google scholar yielded a total of five cases of colorectal cancer after surgery for Hirschsprung disease: two Soave procedures, two modified Duhamel and one ileoanal pull-through [14, 15]. A common feature of these cases was that the tumor mass grew mostly outside the intestinal wall, unlike primary colorectal cancers that arise from polyps. This pattern led to advanced tumor stages with irresectable tumor [15] or peritoneal carcinomatosis originating from a tumor in a post-Soave stricture [14]. Among these five cases, one was deemed irresectable, while four were resectable, with two requiring rectal extirpation. Notably, all reported cases involved mucinous adenocarcinoma, whereas in the present case, pathology revealed moderately differentiated adenocarcinoma.

During the initial rectoscopy, stool remnants were observed in the rectal stump and it was dilated, which could promote inflammation of the mucosa besides diversion proctitis. The risk of diversion colitis associated rectal cancer has been reported to be about 5% for dysplasia and cancer in a median surveillance of 5 years [16]. It is conceivable that a shorter rectal stump could prevent fecal stasis in the stump, thereby reducing the risk of inflammation while also improving continence function [8].

Our patient received the Duhamel procedure during the first decade after its invention and he was comparably young at the time of colorectal cancer diagnosis. Adding this case to the existing cases in literature further raises the question whether the surgical technique itself predisposes patients to colorectal cancer. The cases on rectal cancer after childhood surgery for Hirschsprung disease have emerged after various surgical techniques, with a median interval of approximately 40 years after the index surgery. Hypothetically, not only stool stasis in a rectal stump after Duhamel procedure but the anastomosis itself with local inflammation may predispose for cancer development. A similar phenomenon has been observed in proctocolectomy with ileal pouch-anal anastomosis for ulcerative colitis, where secondary cancer in the cuff region is an unlikely but recognized long-term complication [17]. The near future should clarify this aspect as more patients, that have undergone surgeries for Hirschsprung disease in their childhood, will reach the age at risk of colorectal cancer. If so, this collective should be under consideration for more intense follow-up via colonoscopy or occult blood stool test to prevent advanced colon cancers.

## Conclusion

This case highlights the rare occurrence of colorectal cancer after rectal surgery for Hirschsprung disease, emphasizing the challenges of diagnosis, treatment, and functional outcomes in altered anatomy. It raises the possibility of a cancer predisposition linked to childhood surgeries, particularly the Duhamel procedure besides genetic and lifestyle influences. As more patients after Hirschsprung surgery will reach the age of risk for colorectal cancer, causality might be established. Intensified surveillance of those patients may be necessary to ensure early detection and improved long-term outcomes.

## Data Availability

The data of this case is available upon reasonable request from the corresponding author.
